# Research progress on GlnR-mediated regulation in Actinomycetes

**DOI:** 10.3389/fmicb.2023.1282523

**Published:** 2023-11-22

**Authors:** Bo Gao, Guoqiang Li, Dayong Gu, Jin Wang

**Affiliations:** ^1^Department of Laboratory Medicine, Shenzhen Key Laboratory of Medical Laboratory and Molecular Diagnostics, Shenzhen Institute of Translational Medicine, The First Affiliated Hospital of Shenzhen University, Shenzhen Second People's Hospital, Shenzhen, China; ^2^Guangdong Key Laboratory for Biomedical Measurements and Ultrasound Imaging, National-Regional Key Technology Engineering Laboratory for Medical Ultrasound, School of Biomedical Engineering, Shenzhen University Medical School, Shenzhen, China; ^3^College of Life Science, Northwest A&F University, Yangling, Shaanxi, China

**Keywords:** Actinomycetes, global regulator, GlnR, metabolic regulation, cross-regulation

## Abstract

This review constitutes a summary of current knowledge on GlnR, a global regulator, that assumes a critical function in the regulation of nitrogen metabolism of Actinomycetes. In cross-regulation with other regulators, GlnR was also shown to play a role in the regulation of carbon and phosphate metabolisms as well as of secondary metabolism. A description of the structure of the GlnR protein and of its binding sites in various genes promoters regions is also provided. This review thus provides a global understanding of the critical function played by GlnR in the regulation of primary and secondary metabolism in Actinomycetes.

## 1 Introduction

**Figure 1:**
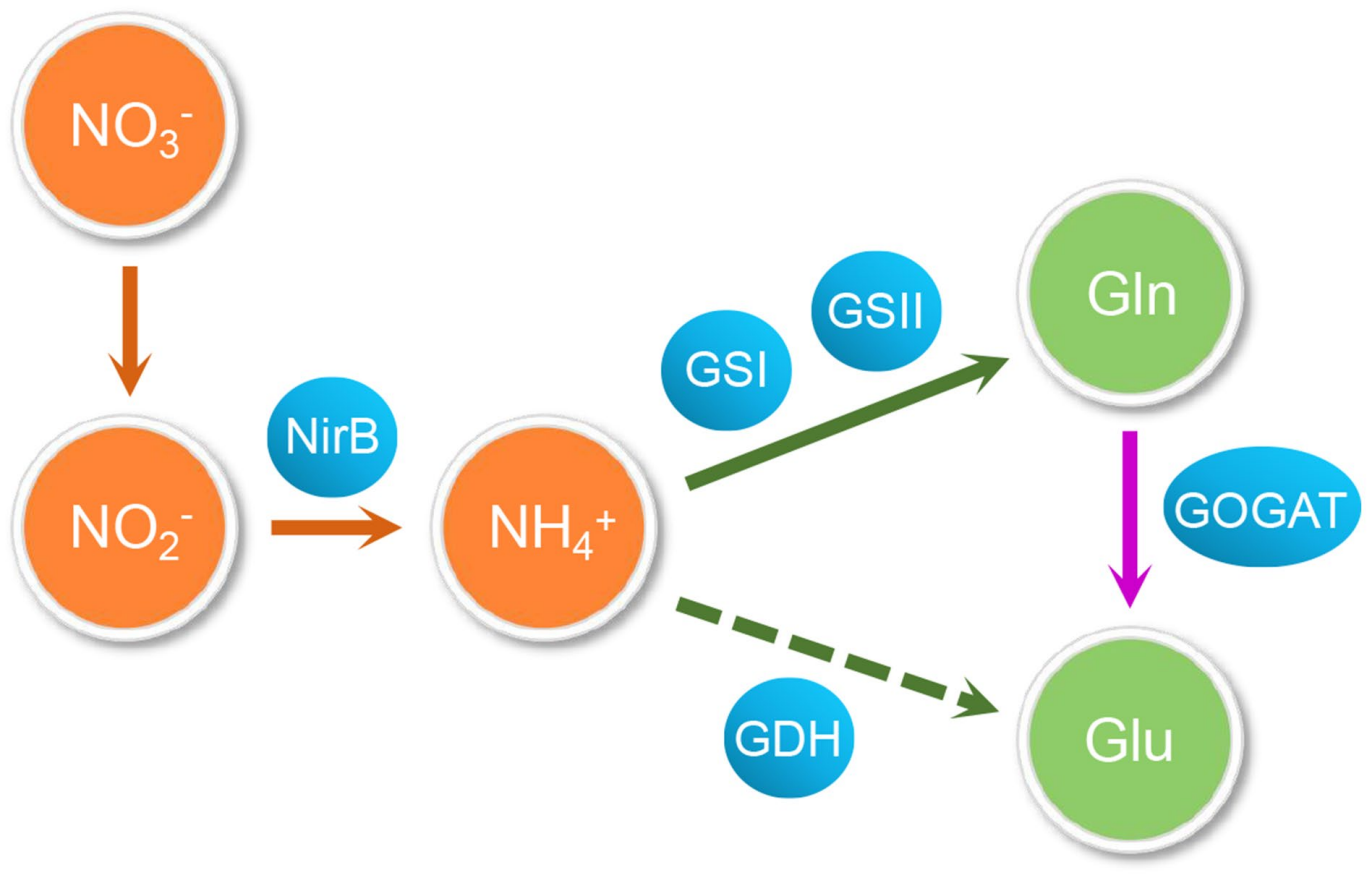
The nitrogen metabolism pathway in actinomycetes. Glutamate and glutamine are synthesized from various nitrogen sources through assimilation and they are the main intracellular nitrogen donors. The glutamate dehydrogenase (GDH) and the glutamine synthetase/glutamate synthetase (GS/GOGAT) systems are two key pathways of ammonium assimilation: When nitrogen supply is sufficient, glutamine is synthesized from ammonia and 2-oxoglutarate through GDH mediates with NADPH consumption; Under low nitrogen concentrations, the GS/GOGAT system consumes ATP to catalyze the formation of glutamine and glutamate. GS converts ammonium and glutamate to form glutamine, and GOGAT catalyzes glutamine and 2-oxoglutarate into two molecules of glutamate.

Both organic and inorganic forms of nitrogen sources are essential for bacterial growth and important components of cellular biomolecules, such as nucleotides, amino acids, and amino sugars (Merrick and Edwards, 1995). Actinomycetes are gram-positive bacteria with a high GC content (Berdy, 2005) that are able to produce, usually late in growth, a great variety of different bio-active molecules such as anti-bacterial, anti-fungi, herbicide, insecticide, immunosuppressants, immunomodulators, and anti-cancer drugs etc. (Berdy, 2005). The biosynthesis of these molecules is regulated by nitrogen, carbon, and phosphate sources availability (Hodgson, 2000; Reuther and Wohlleben, 2007). Among Actinomycetes, *Streptomyces* are major active metabolite producers, able to synthetize a great variety of bioactive metabolites of industrial interest with potential medical and agricultural applications (Ossai et al., 2022). *Streptomyces* are soil-dwelling bacteria with a unique and complicated life cycle, including vegetative mycelium formation, aerial mycelium differentiation, and sporulation (Chater, 2001). This complex morphological differentiation process, that is usually accompanied by the synthesis of bio-active specialized metabolites, involves temporally and spatially regulated regulatory networks (Hopwood et al., 1995). In soil habitats, *Streptomyces* compete with other bacteria for nutrients and especially for nitrogen sources (Reuther and Wohlleben, 2007), that are used to synthesize almost all important biological molecules (Arcondeguy et al., 2001) and also play a crucial role in the regulation specialized metabolism (Reuther and Wohlleben, 2007). Actinomycetes have thus developed complex transcriptional, translational and post-translationnal regulatory systems to regulate the expression of genes and activity of proteins involved in nitrogen metabolism in response to changes in environmental nitrogen levels (Leigh and Dodsworth, 2007). Diverse nitrogen resources, either mineral (nitrate, ammonium, etc.) or organic (urea, glutamine, etc.), are present in the environment. When the bacteria is facing nitrogen organic source shortage in its environment, it can assimilate inorganic nitrogen resources into organic ones (Sun et al., 2017). The two amino acids, glutamate and glutamine, that are the main intracellular nitrogen donors, can be synthesized from various mineral nitrogen sources and especially ammonium (Reitzer and Schneider, 2001; Sun et al., 2017). The nitrogen of glutamate is used for the biosynthesis of numerous amino acids (Fisher, 1989) whereas glutamine is used for the biosynthesis of both purines and pyrimidines, as well as other nitrogenous metabolites (Fisher, 1992). Depending on nitrogen availability, there are two key pathways of ammonium assimilation: the glutamate dehydrogenase (GDH) and the glutamine synthetase/glutamate synthase (GS/GOGAT) pathways (Fisher, 1989; Fisher, 1992) (Figure 1). In *Streptomyces coelicolor*, when the nitrogen supply is high, glutamine is synthesized from ammonium and 2-oxoglutarate by NADPH-dependent GDH whereas under low nitrogen concentrations, the GS of the GS/GOGAT system catalyzes the formation of glutamine from ammonium and glutamate, consuming ATP whereas GOGAT catalyzes the formation of two glutamate molecules from glutamine and 2-oxoglutarate (Magasanik, 1982; Tiffert et al., 2008). Most studies on microbial nitrogen metabolism and its regulatory network come from enterobacter (Magasanik, 1982; Merrick and Edwards, 1995; Oelze and Klein, 1996; Dubbs and Tabita, 2004; Zhang et al., 2014). In gram-negative *Escherichia coli*, ammonium assimilation is regulated by the two-component system NtrB/NtrC (Weiss et al., 2002) that requires σ^54^ to activate the transcription of target genes (Zimmer et al., 2000; Reitzer and Schneider, 2001). When bacteria are starved of nitrogen sources, the kinase NtrB phosphorylates NtrC that stimulates the transcriptional expression of downstream genes involved into nitrogen metabolism (Weiss et al., 2002). In the gram-positive bacteria, *Bacillus subtilis*, with a low GC content, the intracellular nitrogen availability is mainly regulated by two regulators of the MerR family, TnrA and GlnR (GlnR^bsu^) (Schreier et al., 1989; Wray et al., 1996). In excess of nitrogen, GlnR^bsu^ represses the transcription of genes of nitrogen metabolism (Tiffert et al., 2008) whereas in condition of nitrogen limitation, TnrA activates the expression of the *nrgAB*, *nasB*, *gabP*, and *ureA* genes and represses that of *glnRA* (Wray et al., 1996). GlnR senses nitrogen excess indirectly by binding glutamine-feedback-inhibited-GS (FBI-GS). FBI-GS, as a GlnR chaperone, binds the GlnR C-terminal domain within its active-site cavity, promoting the formation of a DNA-binding-competent GlnR dimer and leading to GlnR DNA-binding activation (Travis et al., 2022).

In gram-positive Actinomycetes, with high GC content, the regulation mechanism of nitrogen metabolism evolved completely differently from that of *E. coli* and *B. subtilis* (Burkovski, 2003). The nitrogen metabolism of most Actinomycetes (such as *S. coelicolor*, *Amycolatopsis mediterranei*) is mediated by GlnR (Amon et al., 2009), a regulator of the OmpR family that is an orphan response regulator, significantly different from GlnR^bsu^ of *B. subtilis* (Hutchings et al., 2004). In contrast, a small number of Actinomycetes (such as *Mycobacterium smegmatis*, *Streptomyces avermitilis*) bear two regulatory proteins, GlnR and AmtR, involved in the regulation of nitrogen metabolism whereas only AmtR regulates nitrogen metabolism in *Corynebacterium* (Burkovski, 2003; Beckers et al., 2005). In 1988, the *glnA* gene encoding glutamine synthase GSI was located in *S. coelicolor,* and GSI enzyme activity was determined (Wray and Fisher, 1988). In the 1990s, the research mainly focused on GlnR and GSI/GSII. In 1990, a second gene, *glnII,* encoding glutamine synthase GSII was identified in *Frankia* and *Streptomyces viridochromogenes* (Behrmann et al., 1990; Rochefort and Benson, 1990). In 1991, the isolation of six glutamine-deficient mutants of *S. coelicolor* led to the identification of the *glnR* encoding gene whose transcription start sites were determined (Wray et al., 1991). In 1993, the positive role that GlnR plays in the regulation of *glnA* expression was demonstrated (Wray and Fisher, 1993) as well as the post-translational modification of GSI (Fisher, 1992). With the completion of the genome sequence of *S. coelicolor* in 2002 and that of other *Streptomyces* subsequently, the conserved GlnR binding sites were identified (Tiffert et al., 2008) and used to identify GlnR target genes. Moreover, cross-regulation between GlnR and other transcription regulators was demonstrated (Rodriguez-Garcia et al., 2007; Yang et al., 2009; Wang R. et al., 2013). Since 2000, the functional identification of GlnR was achieved in other Actinomycetes (such as *A. mediterranei* U32, *M. smegmatis*, *Saccharopolyspora erythraea*, *Streptomyces* var*iabilis*). In 2011, Tiffert et al. performed a proteomic analysis of the *glnR* deletion mutant of *S. coelicolor* and found that GlnR affects the expression of numerous genes belonging to different metabolic pathways (Tiffert et al., 2011).

This review focuses on the functional studies of GlnR mainly in *S. coelicolor* but also in other antibiotic producing *Streptomyces* species as well as in other Actinomycetes. This review reports what is known on the role played by GlnR in the regulation of nitrogen as well as of carbon and phosphate metabolisms and on the cross-regulation between GlnR and other regulators. The structure of the protein GlnR and of that of its target sites is also commented. This review thus provides a systematic understanding of the critical role played by the global regulator GlnR in the regulation of both primary and specialized metabolism in Actinomycetes.

## 2 Regulatory role and regulatory targets of GlnR in *Streptomyces coelicolor* and other Actinomycetes

Tiffert et al. performed a comparative proteome analysis of the wild type strain of *S. coelicolor* M145 and a *glnR*-deficient mutant and discovered that GlnR regulates the expression of numerous proteins (Tiffert et al., 2011) involved (1) amino acid metabolism, GlnR inhibits the biosynthesis and degradation of certain types of amino acids (methionine, tryptophan, serine), (2) carbon metabolism, GlnR enhances the synthesis of acetyl-CoA and inhibits the pentose phosphate pathway, (3) stress response, GlnR promotes the transcription of numerous stress-response genes (Tiffert et al., 2011), and (4) antibiotic biosynthesis. Whether GlnR directly or indirectly affects these processes requires further experimental demonstration, but it is clear that GlnR plays a pleiotropic role in the regulation of *S. coelicolor* M145 metabolism.

### 2.1 In nitrogen metabolism

#### 2.1.1 In *Streptomyces coelicolor*

In *S. coelicolor* as well as in other *Streptomyces* nitrogen metabolism is mainly regulated by the regulator GlnR. However a second GlnR-like regulator was identified and called GlnRII. GlnR and GlnRII share 31% identity (Fink et al., 2002). GlnRII can interact with the promoter region of some genes of nitrogen metabolism, such as *glnA*, *glnII*, and *amtB*, but differ from GlnR in its physiological function since a mutant deleted for the *glnRII* gene does not behave as a *glnR* mutant (Reuther and Wohlleben, 2007). When nitrate is used as sole nitrogen source, the *glnR* mutant of *S. coelicolor* exhibits glutamine-auxotrophy but can survive with extremely low amounts of ammonium uptake and assimilation but that is not the case of the *glnRII* mutant (Tiffert et al., 2008). GlnR is thus the primary regulator of nitrogen metabolism and GlnRII may promote cell differentiation and antibiotic production under nitrogen limitation (Fink et al., 2002). GlnR regulates the expression of many essential genes involved into nitrogen metabolism, including nine genes directly involved into nitrogen metabolism (Table 1): *amtB*-*glnK*-*glnD* operon (encoding a putative ammonium transporter and nitrogen signaling proteins), *nasA* (encoding a putative nitrate reductase), *nirB* (encoding a putative nitrite reductase), *ureA* (encoding urease), *glnA* and *glnII* (encoding glutamine synthetase), *gdhA* (encoding glutamate dehydrogenase); and seven other genes indirectly involved in nitrogen assimilation: the putative transcriptional regulator SCO0255, the putative NADPH-dependent FMN reductase SCO0888, the putative membrane protein SCO2400, the putative sugar-binding protein SCO2404 of an ABC transporter, and three other hypothetical proteins of unknown function: SCO1863, SCO2195, SCO7155 (Tiffert et al., 2008). The nitrogen metabolism genes/operons (*glnQHMP*, *nasD1*, *nasD2EF, ansZ*, *pucR*, *gcvH*, *oppABC, dppABC*, and *appABCDF*) were also recently shown to belong to the GlnR regulon (Wang T. S. et al., 2023). In nitrogen-limited environments, GlnR positively regulates the expressions of *glnA*, *glnII*, *amtB*, *nirB*, and *nasA* (Tiffert et al., 2008; Wang and Zhao, 2009; Qiu et al., 2022) and negatively regulates the expressions of *gdhA*, *ureA*, *sco0255*, *sco0888*, *sco2400*, and *sco2404* (Tiffert et al., 2008). Glutamine synthetase (GS) is crucial in ammonium assimilation, and many bacteria have GS isoenzymes (Murray et al., 2013). *S. coelicolor* possesses two types of GS, GSI encoded by *glnA* and GSII encoded by *glnII* (Wray and Fisher, 1988; Behrmann et al., 1990). In addition to the *glnA^sco^* (also named as *glnA1^sco^*), there are three *glnA*-type genes *glnA2^sco^* (encoding γ-glutamylpolyamine synthetase), *glnA3^sco^* (encoding γ-glutamylpolyamine synthetase), and *glnA4^sco^* (encoding γ-glutamylethanolamide synthetase), which are not directly involved in L-glutamine synthesis and nitrogen assimilation (Rexer et al., 2006; Krysenko et al., 2022). It has been demonstrated that the first step in polyamine catabolism is catalyzed by GlnA2^sco^ (Krysenko et al., 2022). The GlnA3^sco^ and GlnA4^sco^ catalyze the first step in poly−/monoamine assimilation, respectively (Krysenko et al., 2021). GSI and GSII are homomultimeric proteins composed of 12 and 10 identical subunits, respectively (Weissschuh et al., 2000; Zeth, 2013). The expression of GSI is positively regulated by GlnR at the transcriptional level and the activity of the GSI enzyme is regulated post-translationally by adenylylation by the adenylyltransferase GlnE (Fisher and Wray, 1989). Under nitrogen limitation, GSI is activated, whereas under nitrogen excess, GSI is adenylylated by GlnE and GSI-AMP is inactive (Hesketh et al., 2002). In contrast, GSII is not modified by adenylylation (Brown et al., 1994). The GlnE-dependent modulation of GSI activity in *S. coelicolor* obviously does not require the GlnK/GlnD system (Hesketh et al., 2002). In *S. coelicolor*, GlnR also positively regulates the transcriptional expression of *nnaR* (nitrate/nitrite assimilation regulator) and NnaR activates directly the transcriptional expression of 4 target genes: *narK* (encoding a putative nitrate extrusion protein), *nirB* (encoding a putative nitrite reductase), *nirA* (encoding a putative nitrite/sulfite reductase), and *nasA* (encoding a putative nitrate reductase) (Amin et al., 2012). NnaR cooperatively binds the *nirB* promoter together with GlnR, indicating that NnaR may function as a co-activator of GlnR and participate in the transcriptional control of nitrate/nitrite assimilation genes (Amin et al., 2012). The regulatory role of NnaR in nitrate and nitrite assimilation had also been demonstrated in mycobacteria (Antczak et al., 2018).

**Table 1:** GlnR gene targets involved in nitrogen metabolism.

Gene	Annotation	*S. coelicolor* homolog	References
*glnA*	Glutamine synthetase Ι	SCO2198	Wray and Fisher (1988)
*glnII*	Glutamine synthetase II	SCO2210	Rochefort and Benson (1990) and Behrmann et al. (1990)
*glnRII*	Nitrogen response regulator	SCO2213	Fink et al. (2002)
*amtB*	Putative ammonium transporter	SCO5583	Tiffert et al. (2008) and Wang et al. (2012)
*glnK*	PII signal protein	SCO5584	Tiffert et al. (2008)
*glnD*	Adenylyl transferase	SCO5585	Tiffert et al. (2008)
*nirA*	Putative nitrite/sulfite reductase	SCO6102	Amin et al. (2012)
*nirB*	Putative nitrite reductase	SCO2486	Tiffert et al. (2008) and Amin et al. (2012)
*nasA*	Putative nitrate reductase	SCO2473	Wang and Zhao (2009) and Amin et al. (2012)
*ureA*	Urease	SCO1236	Tiffert et al. (2008)
*gdhA*	Glutamate dehydrogenase	SCO4683	Tiffert et al. (2008)
*nnaR*	Nitrate/nitrite assimilation regulator	SCO2958	Tiffert et al. (2008) and Amin et al. (2012)
*narK*	Putative nitrate extrusion protein	SCO2959	Amin et al. (2012)
*nasN*	Nitrate reductase	Unassigned	Tan et al. (2020)
*narGHJI*	Nitrate reductase	Unassigned	Malm et al. (2009)
*nirBD*	Nitrite reductase	SCO2487	Malm et al. (2009)
*acuA/srtN*	Acetyltransferase/deacetylase	Unassigned	You et al. (2016, 2017)
*nasACKBDEF*	Nitrate assimilation	Unassigned	Shao et al. (2011)
*gltBD*	Glutamate synthase	SCO2026	Yoshida et al. (2003)
*gltP*	Na(+)/glutamate: H(+) symporter	SCO4498	Yoshida et al. (2003)
*glnQ*	ATP-binding protein of a glutamate ABC-type transporter	SCO2831	Yao et al. (2014)
Unassigned	Putative transcriptional regulator	SCO0255	Tiffert et al. (2008)
Unassigned	Putative NADPH-dependent FMN reductase	SCO0888	Tiffert et al. (2008)
Unassigned	Putative membrane protein	SCO2400	Tiffert et al. (2008)
Unassigned	Putative sugar-binding protein of an ABC transporter	SCO2404	Tiffert et al. (2008)
Unassigned	Hypothetical protein	SCO1863	Tiffert et al. (2008)
Unassigned	Hypothetical protein	SCO2195	Tiffert et al. (2008)
Unassigned	Hypothetical protein	SCO7155	Tiffert et al. (2008)

#### 2.1.2 In mycobacteria

*M. smegmatis*, *Mycobacterium tuberculosis*, *Mycobacterium leprae*, and *Mycobacterium bovis* are the most commonly studied *Mycobacterium* genus (Ergan et al., 2004; Amon et al., 2009; Schuenemann et al., 2013; Meehan et al., 2019; European Food Safety Authority and European Centre for Disease Prevention and Control, 2021). Different from *S. coelicolor*, *glnA1^myc^, glnA2^myc^, glnA3^myc^*, and *glnA4^myc^* are all encoding GS genes present in *M. tuberculosis* (Rakovitsky et al., 2018). In all mycobacteria, the GS genes *glnA1^myc^* and *glnA2^myc^* are present and have conserved genomic positions near *glnE*. The GS genes *glnA3^myc^* and *glnA4^myc^* are present in most mycobacteria but not in *M. leprae* (Amon et al., 2010). The model strain, *M. smegmatis*, bears at least three orthologues of GlnA4^myc^ (Amon et al., 2010). The gene *gdhA* is only present in *M. smegmatis* whereas this gene is absent in other mycobacteria that assimilate ammonium through GS/GOGAT pathway (Amon et al., 2010). *M. smegmatis* bears three putative ammonium transporters: AmtB, Amt1, and AmtA (Amon et al., 2009). The *amtB* forms an operon with *glnK* and *glnD*, as in *Streptomyces*. The *amt1* gene is present in a cluster of three genes encoding glutamine synthetase, glutamine amidotransferase, and glutamate synthase, whereas *amtA* is monocistronic (Amon et al., 2009). As *glnA*, the *amtB-glnK-glnD* and *amt1* operons are positively controlled by GlnR (Amon et al., 2008). Genomics analysis of *M. smegmatis*, identified GlnR as the global nitrogen response regulator. It directly controls the transcription of over 100 genes involved in crucial nitrogen stress survival processes, such as nitrate and urea utilization and the use of cellular components as a source of ammonium (Jenkins et al., 2013). Moreover, GlnR controls redox sensing and lipid anabolism through WhiB3 (You et al., 2019) and controls cholesterol catabolism through KstR (Ma et al., 2022). *M. smegmatis* also has another global nitrogen regulator, AmtR, whose expression is controlled by a *cis*-encoded small RNA (Petridis et al., 2016) and competes with GlnR to regulate urea metabolism. Furthermore, the nitrate reductase NasN, is required for growth when nitrate is the only nitrogen source available, and GlnR regulates *nasN* expression (Tan et al., 2020).

In *M. tuberculosis*, NarGHJI (nitrate reductase) and NirBD (nitrite reductase) mediate the assimilatory reduction of nitrate and nitrite and GlnR activates *nirBD* transcription (Malm et al., 2009). Nitrate is present in infected tissues (Bogdan, 2001) and nitrate assimilation by *M. tuberculosis* is essential for its survival *in vitro* and *in vivo* (Hedgecock and Costello, 1962). Under nitrogen limitation, GlnR controls the expression of at least 33 genes (Malm et al., 2009). In contrast to non-pathogenic mycobacteria, in *M. tuberculosis* GlnR regulates the expression of genes associated with nitric oxide detoxification and intracellular survival (Williams et al., 2015). Although *M. tuberculosis* has four genes encoding glutamine synthetases (GS), mainly GlnA1 is highly expressed and is a critical enzyme for growth both *in vitro* and *in vivo* (Harth et al., 2005). In addition, GlnA1 is critical for *M. tuberculosis* virulence and may be a potential drug target for tuberculosis treatment (Tullius et al., 2003). In most mycobacteria, GlnR activates its own transcription in response to nitrogen-limitation (He et al., 2023). The auto-regulation of *glnR* transcription is ubiquitous in other mycobacterial species and most mycobacteria *glnR* promoter regions have potential GlnR binding sites (Shi et al., 2023). The study also demonstrated that the purified GlnR protein of *M. smegmatis* can precisely attach 16 promoter regions present in different mycobacteria species (He et al., 2023).

#### 2.1.3 In Pseudonocardiaceae

Pseudonocardiaceae, belonging to Actinobacteria genera, are able to degrade cellulose and to produce antibiotics (Carr et al., 2012; Cuesta et al., 2013; Mast et al., 2020).

In *S. erythraea*, 25 genes related to nitrogen utilization regulated by GlnR were identified as associated with ammonium uptake and assimilation, urea utilization, nitrite/nitrate assimilation, glutamate transport, arginine biosynthesis, nitric oxide biosynthesis, etc. (Yao et al., 2014). The GlnR-mediated regulation of the GDH and GS/GOGAT pathways of nitrogen metabolism is similar to that of *Streptomyces*, ammonium is the preferred nitrogen source of *S. erythraea* (Yao et al., 2014). In ammonium-rich conditions, the inhibition of *gdhA* expression by GlnR is relieved, and the GDH pathway is primarily used for ammonium assimilation. In condition of low ammonium concentration, the expression of GS gene and GOGAT (glutamine oxoglutarate aminotransferase) gene controlled by GlnR are significantly upregulated. Additionally, two of the three putative urease-encoding operons are positively controlled by GlnR (Yao et al., 2014). As TnrA, the global regulator of nitrogen metabolism of *B. subtilis* (Yoshida et al., 2003), GlnR directly regulates the expression of *gltBD* operons (encoding GOGAT), *gltP* genes (encoding Na(+)/glutamate: H(+) symporters), and *glnQ* (encoding the ATP-binding protein of a glutamate ABC-type transporter). Unlike *Streptomyces*, *S. erythraea* has no homolog of *glnD*, so one ignores how GlnK activity is regulated in this strain (Yao et al., 2014). The expression of the acetyltransferase AcuA is positively regulated by GlnR and AcuA acetylates GlnA1 and GlnA4 in *S. erythraea*. This lysine acetylation inactivates GlnA4 but does not affect GlnA1 activity. The acetylated GlnA1 enhances GlnR-DNA binding, and this regulatory effect of acetylated GlnA1 is highly conserved in Actinomycetes (You et al., 2016).

In *A. mediterranei* U32, GlnR positively regulates *glnA*, *amtB* and the *nas* operon in conditions of nitrogen limitation (Yu et al., 2006; Wang Y. et al., 2013). However, under different nitrogen source conditions, there is no linear relationship between the amount of GlnR protein and *glnA* expression in U32 (Yu et al., 2007). In addition, the activity of GDH does not change with nitrogen sources, so its encoding gene *gdhA* might not be regulated by GlnR (Wang et al., 2014). Alanine dehydrogenase (AlaDH) has also been confirmed to be associated with ammonium assimilation in U32 under nitrogen-rich conditions (Wang et al., 2014). Notably, the AlaDH pathway is the major route alternative to the GDH pathway under high ammonium conditions (Ni et al., 1984). At the same time, GlnR represses the transcription of the AlaDH encoding gene, *ald*, under nitrogen limitation (Wang et al., 2014). Furthermore, NasE, a homolog of NnaR in U32, also affects nitrate/nitrite assimilation (Shao et al., 2011).

### 2.2 In carbon metabolism

Consistently with the proteomic data from Tiffert’s study, GlnR was shown to regulate carbon metabolism (Liao et al., 2014, 2015; Cen et al., 2016; You et al., 2017; Wang T. S. et al., 2023). In Actinomycetes, GlnR was shown to directly regulate carbon source up-take of non-phosphotransferase-system (non-PTS) via its binding to promoter region of 13 of the 20 carbohydrate ATP-binding cassette (ABC) transporters encoded genes present in the genome of these bacteria (Liao et al., 2015). GlnR-mediated regulation of non-PTS carbon source utilization is highly conserved in *S. coelicolor*, *M. smegmatis*, and *S. avermitilis*, indicating the importance of GlnR for the regulation carbohydrate metabolism in Actinomycetes (Liao et al., 2015). In the latest research, carbon metabolism genes *ldh3* (encoding lactate dehydrogenase) and *maeA1* (encoding malic enzyme) are newly identified targets of GlnR (Wang T. S. et al., 2023).

#### 2.2.1 In *Streptomyces*

In *S. coelicolor*, the expression of the *agl3EFGXYZ* operon encoding a putative ABC-type carbohydrate transporter, noted as a putative multiple-sugar transporter (Sprusansky et al., 2003), is repressed by both Agl3R, a regulator of the GntR-family but also by GlnR (Cen et al., 2016). It is only in condition of low carbon and high nitrogen availability that the transcription of the *agl3EFGXYZ* operon is derepressed, when both GlnR and Agl3R are inactivated (Cen et al., 2016). Since GlnR regulated both carbon and nitrogen metabolisms, overexpressing GlnR can improve the utilization of less preferred carbon sources (Liao et al., 2015). Furthermore GlnR inhibits the expression of the *ect* biosynthesis cluster (*ectABCD*) (Shao et al., 2015), that is widespread in microorganisms and directs the synthesis of ectoine and hydroxyectoine that plays a role is the resistance to osmotic stress and high temperature (Bursy et al., 2008; Kol et al., 2010). Since ectoine and hydroxyectoine production requires the consumption of glutamate, bacteria maintain intracellular glutamate concentration by inhibiting the transcription of *ect* biosynthesis cluster by GlnR when nitrogen availability is low (Shao et al., 2015). This function of GlnR has also been confirmed in *A. mediterranei* U32 and *S. avermitilis* (Shao et al., 2015), suggesting the possible of general function of GlnR to regulates osmotic pressure in Actinomycetes.

#### 2.2.2 In mycobacteria

In *M. smegmatis* GlnR controls negatively the glyoxylate and methylcitrate cycles by directly inhibiting the transcription of the *icl* gene (encoding isocitrate lyase) (Qi et al., 2021) and the *prpDBC* operon (*prpD* encoding methylcitrate dehydratase, *prpB* encoding methylisocitrate lyase, and *prpC* encoding methylcitrate synthase) (Liu et al., 2019). In *Mycobacterium neoaurum*, GlnR also represses the expression of the *prp* operon in condition of low nitrogen availability so in a *ΔglnR* mutant strain the *prp* operon is overexpresses and this strain efficiently produces androstenedione (Zhang et al., 2020).

#### 2.2.3 In *Saccharopolyspora*

GlnR also shown to regulate the expression of genes involved in carbon metabolism in *S. erythraea*. GlnR enhances the synthesis of acetyl-CoA via the direct activation of the transcription of genes encoding acetyl-CoA synthetases (*acsA1*, *acsA2*, and *acsA3*). Interestingly, the activities of the three acetyl-CoA synthetases are regulated post-translationally via acetylation of a lysine residue by the couple “protein acetyltransferase AcuA/deacetylase SrtN” (You et al., 2017). In response to unknown signals of nitrogen starvation, GlnR regulates acetate metabolism, thereby coordinating nitrogen and carbon metabolism (You et al., 2017). In *S. erythraea* GlnR also negatively controls the expression of the citrate synthase encoding genes *citA* and *citA4* whereas its binding to the promoter region of another citrate synthase encoding gene *gltA-2* has no impact on the level of expression of this gene (Liao et al., 2014).

### 2.3 In specialized metabolites biosynthesis

The regulatory effect of GlnR on antibiotic synthesis was analyzed in different *Streptomyces* species. In *S. coelicolor*, GlnR was directly associated with the biosynthesis of two antibiotics, actinorhodin and undecylprodigiosin, via the pathway-specific activator genes *actII-ORF4* and *redZ*, respectively (He et al., 2016). In *Streptomyces hygroscopicus*, GlnR regulates the expression of the validamycin A (a C_7_N-aminocyclitol antibiotic) biosynthesis gene clusters via its direct binding to the *valK-valA-int* promoter region (Qu et al., 2015). In *S. avermitilis*, GlnR could stimulate avermectin production directly through the binding to the respective pathway-specific activator encoding genes, *aveR* and *olmRI/RII*, and the binding motif of GlnR was determined (Table 2) (He et al., 2016).

**Table 2:** The conserved GlnR box in actinomycetes.

Strain	GlnR box	References
*Streptomyces coelicolor*	gTnAc-n_6_-GaAAc	Tiffert et al. (2008)
*Streptomyces venezuelae*	GTnAC-n_6_-GTnAC	Pullan et al. (2011)
*Streptomyces avermitilis*	GAAAC-n_6_-GTATC	He et al. (2016)
*Mycobacterium smegmatis*	t/gTAAC-n_6_-Gc/aAAC	Amon et al. (2008)
*Saccharopolyspora erythraea*	t/gna/cAC-n_4_cn-GnAAc	Yao et al. (2014)

In *Streptomyces lincolnensis*, GlnR directly regulates the biosynthesis of lincomycin by enhancing the expression of nitrate-specific ABC transporter genes, nitrate assimilation genes, and lincomycin transporter LmrA (Meng et al., 2017). GlnR is upregulated when nitrate is provided as nitrogen source (Wang et al., 2022) and positively regulates the transcription of the lincomycin transporter LmrA (Meng et al., 2017) whereas RamR as well as AflQ1-Q2 negatively regulate lincomycin biosynthesis and promote morphological development (Wang et al., 2022). Therefore, the disruption of *aflQ1-Q2* is likely to lead to an increase of lincomycin production (Wang R. D. et al., 2023) but not that of RamR that was shown to directly positively regulates GlnR expression (Wang et al., 2022).

In *A. mediterranei* U32, GlnR also regulates positively rifamycin production GlnR via the direct activation of the expression of the regulators RifZ and RifK that activate the transcription of genes of the *rif* cluster (Liu et al., 2020). Interestingly, the over-expression of GlnR of U32 in *S. coelicolor* led to a decrease of actinorhodin production and an increase of undecylprodigiosin production (Yu et al., 2007).

**Figure 2:**
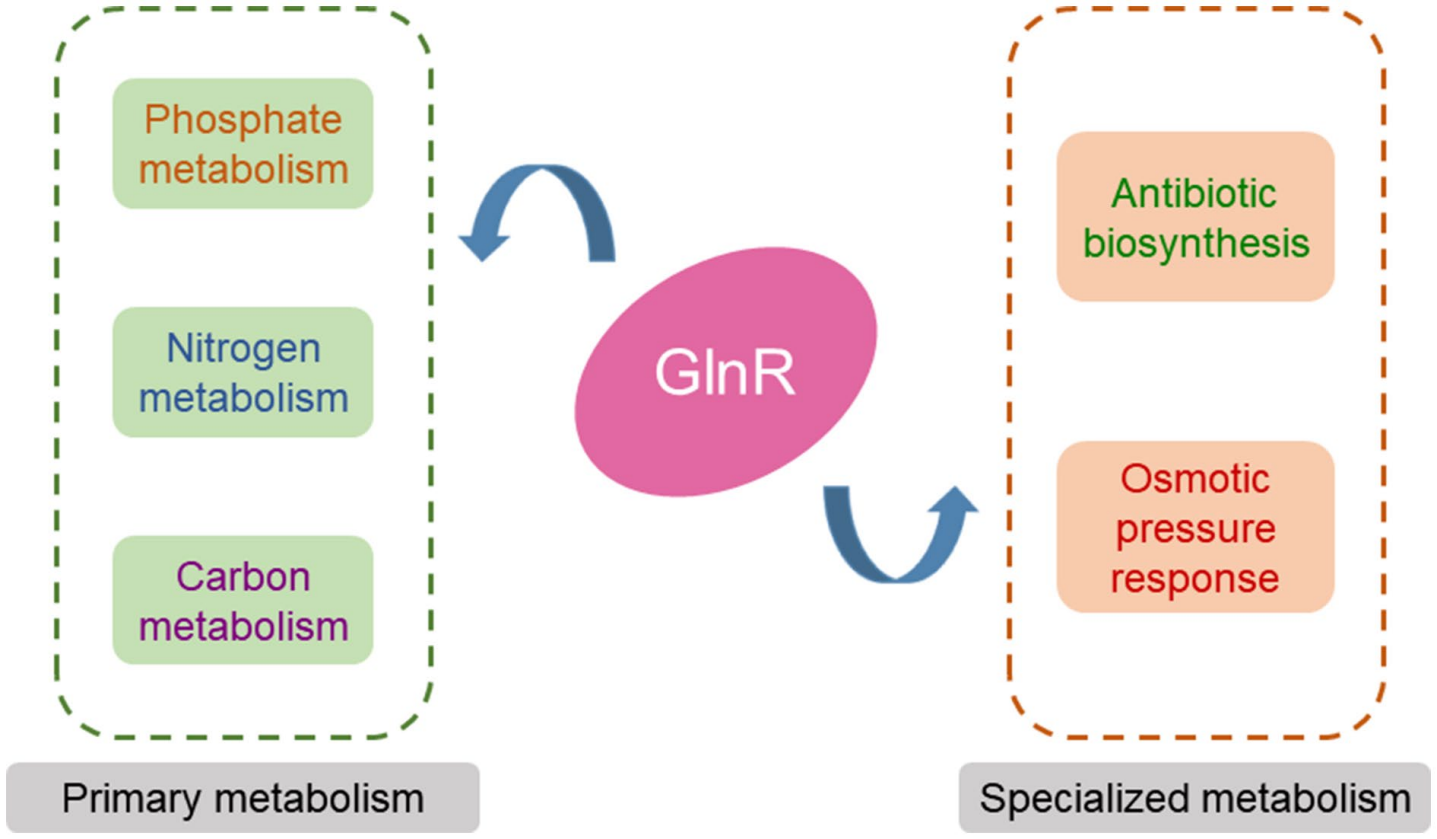
The GlnR regulatory network in actinomycetes. GlnR regulates nitrogen metabolism, phosphate metabolism, carbon metabolism, antibiotic biosynthesis, and the response of bacteria to osmotic pressure in actinomycetes.

In conclusion, GlnR is a global regulator in actinomycetes that can affect not only nitrogen but also phosphate and carbon metabolisms, the adaptation to high osmotic pressure as well as antibiotic biosynthesis (Figure 2).

## 3 Cross-regulation between GlnR and other regulators

The essential nutrients, such as carbon, nitrogen, and phosphorus, usually limit the growth of soil-dwelling bacteria. The deficiency of a particular nutrient will trigger a series of interrelated reactions governed by specific regulators to maintain metabolic balance (Martin et al., 2011).

### 3.1 With PhoP and AfsR

In *S. coelicolor*, the relationship between the phosphate and nitrogen metabolic pathways is intricate since PhoP was shown to directly negatively regulates the expression of *glnR*, *glnA*, *glnII*, and *amtB-glnK-glnD* operon (Rodriguez-Garcia et al., 2007, 2009). PhoP and GlnR compete for the binding to the *glnA*, *glnII* and *amtB* promoter regions since their binding sites overlap in these regions (Wang et al., 2012; Sola-Landa et al., 2013). Additionally, PhoP binds to other nitrogen-regulated genes: *ureA* (urease gamma subunit), *sco0255* (putative transcriptional regulator), and *sco1863* (hypothetical protein) (Sola-Landa et al., 2013) but does not bind to the *glnRII* promoter region, indicating another PhoP-independent regulatory pathway exists to control nitrogen metabolism (Rodriguez-Garcia et al., 2009). In contrast, GlnR cannot bind to several important genes of the Pho regulon such as *pstS* and *phoRP* (Martin et al., 2011). This cross-regulation between phosphate and nitrogen metabolism in *S. coelicolor* is thus not reciprocal (Martin et al., 2011). However, interestingly PhoP also binds specifically to the promoter region of *afsS*, a target gene of the SARP-like regulator AfsR that controls antibiotic synthesis (Santos-Beneit et al., 2011). AfsR, can bind to the promoter regions of PhoP-regulated genes such as *afsS*, *pstS*, and *phoRP* (Lee et al., 2002; Santos-Beneit et al., 2009). Furthermore, both PhoP and AfsR can bind to the *glnR* promoter region with overlapping binding sites but have different regulatory effects. PhoP negatively regulates *glnR* expression in condition of phosphate limitation whereas the regulatory effect of AfsR depends on the stage of the culture. AfsR activates *glnR* expression at early growth stages and inhibits it at later growth stages (Santos-Beneit et al., 2012).

In *S. erythraea*, the regulation between GlnR and PhoP is reciprocal, and GlnR can bind to the *phoRP* promoter region to regulate negatively *phoRP* transcription (Yao and Ye, 2016). Moreover, in contrast to *S. coelicolor,* PhoP has a positive rather than a negative role in the regulation of GlnR expression (Yao and Ye, 2016). It was also confirmed that in *S. erythraea*, GlnR and PhoP could synergistically or competitively activate genes associated with starch-degrading enzymes and cellulose-degrading enzymes: *amlB* (encoding an α-amylase), *glaA* (encoding a glucoamylase) and a β-glucosidase-encoding gene (Xu and Ye, 2018), and four genes encoding possible α-glucosidases *aglA2*, *aglA3*, *aglA4*, and *aglA5* (Xu et al., 2016). Therefore, in response to changes in nutrient supplies, bacteria can coordinate the balance between carbon, nitrogen, and phosphate metabolisms through the regulation of polysaccharide degradation mediated by GlnR and PhoP. In addition, Ye et al. demonstrated that PhoP modulates erythromycin biosynthesis in *S. erythraea* by integrating phosphate/nitrogen signals, governing the interaction between phosphate/nitrogen metabolism and specialized metabolism (Xu et al., 2019). Furthermore, GlnR and PhoP enhance the production of erythromycin by regulating primary metabolism and thus precursors availability (Pei et al., 2022).

### 3.2 With MtrA

The regulator MtrA regulates nitrogen and phosphate metabolisms in a GlnR-dependent manner in several *Streptomyces* species (Zhang et al., 2017; Zhu et al., 2019, 2020, 2021, 2022). In some instances, MtrA competes with GlnR to control the expression of genes of the nitrogen metabolism but GlnR plays a decisive role in the control of nitrogen metabolism genes (Zhu et al., 2022). MtrA also regulates morphological differentiation and antibiotic production and its impact on antibiotic production is generally stronger than that of GlnR (Zhu et al., 2020).

### 3.3 With AfsQ1

In *S. coelicolor*, the regulator AfsQ1 of the two-component system AfsQ1/AfsQ2 directly interacts with the promoter regions of seven nitrogen assimilatory genes: *gdhA*, *nirB*, *nasA*, *amtB*, *glnA*, *ureA* and *glnII* but regulates negatively the expression of *amtB*, *glnA*, *ureA*, and *glnII* but not that of *gdhA*, *nirB* and *nasA* in a glutamate-based minimum medium (Wang R. et al., 2013). This finding is consistent with previous report of Nieselt et al. (2010) that indicated that in a medium containing excess of glutamate, the genes related to nitrogen metabolism are negatively regulated by other regulators besides PhoP/PhoR (Nieselt et al., 2010). AsfQ1, together with GlnR, also directly regulate the production of the specialized metabolites actinorhodin and undecylprodigiosin through their pathway-specific activators, *actII-ORF4* and *redZ* (He et al., 2016). Interestingly, in *Streptomyces venezuelae*, the conserved binding site of AfsQ1 does not overlap the previously reported conserved binding site of GlnR (Pullan et al., 2011).

### 3.4 With NdgR and ScbR

In *S. coelicolor*, NdgR regulates nitrogen metabolism, morphological differentiation and antibiotic production. In minimal media containing Leu or Gln, *ndgR* deletion mutant of *S. coelicolor* showed a slow growth rate, a defective morphological differentiation and an altered antibiotic production (Yang et al., 2009). NdgR is unable to directly interact with the promoter regions of the nitrogen metabolism genes *glnR*, *glnA*, and *glnII*, as well as with the promoter region of the antibiotic pathway-specific activator genes *actII-ORF4* and *redD* but it can directly activate the transcription of *scbR* (Yang et al., 2009) that regulates the expression of numerous genes, including antibiotic biosynthetic genes. Furthermore, ScbR can specifically interact with the *glnR* promoter region. Therefore, NdgR may indirectly regulate nitrogen metabolism and antibiotic synthesis through ScbR (Yang et al., 2009). NdgR is an IclR-like regulator, and these regulators have typical ligand binding domains at their C-terminal end (Molina-Henares et al., 2006). The interaction of NdgR with these ligands, that remain to be characterized, may affect its binding ability to its target genes.

In *Streptomcyes peucetius* NdgR is a homolog of AreB of *Streptomyces clavuligerus* that modulates leucine biosynthesis and antibiotic production (Santamarta et al., 2007). In *S. peucetius*, NdgR can directly specifically interact with the promoter regions of doxorubicin biosynthetic genes and regulate antibiotic production (Yang et al., 2009).

In conclusion, the *ndgR* gene that is highly conserved in *Streptomcyes* species and other genetically related bacteria, such as mycobacteria and *Corynebacteria* (Yang et al., 2009), connects primary metabolism to morphological differentiation and antibiotic production. The cross-regulations mentioned above between GlnR, PhoP, MtrA, AfsR, AfsQ1, NdgR and ScbR are regulating primary and secondary metabolisms to maintain the intracellular balance between nitrogen, phosphate and carbon to adjust the bacterial metabolism to the changes in the surrounding environment.

## 4 Protein structure of GlnR

In Actinomycetes, GlnR belongs to the OmpR family of proteins (Amon et al., 2010). OmpR-like proteins are response regulators of the bacterial two-component system. They are primarily transcriptional activators with typical DNA domain at the C-terminal and a signal-receiving domain at the N-terminal end of the protein (MartinezHackert and Stock, 1997). The comparison of GlnR amino acid sequences of 10 Actinomycetes, with the exception of that of *Corynebacterium glutamicum*, revealed that the GlnR proteins share 62 to 82% similarity. The DNA binding domain (α2-loop-α3) was relatively conserved and the α3 sequence, was identical (Tiffert et al., 2008). OmpR-like proteins typically contain a conserved Asp residue as their specific phosphorylation site (Delgado et al., 1993; Lee et al., 2002) whereas two residues, serine/threonine and tyrosine, are involved in phosphate transfer (Hengge, 2001). The OmpR crystal structure of *E. coli* shows that the α2-loop-α3 of the DNA binding domain forms the helix-turn-helix (HTH), where the α3 helix recognizes the specific DNA sequence through direct interaction with the major groove of DNA (Gross et al., 1989). The secondary structure of OmpR of *E. coli* showed that the conserved site of phosphorylation is an Asp residue (D-55) (Delgado et al., 1993), the serine/threonine residue is a Thr residue (T-83) and a tyrosine residue is present as Y-102 (Birck et al., 1999).

In *S. coelicolor*, GlnR is encoded by the *glnR* gene, which has three promoters: P1, P2, and P3. P2 has an identified −10 region, but P1 has no clearly identified −10 region (Wray and Fisher, 1993). The P2 promoter of *glnR* is preferentially recognized by a σ^31^-containing RNA polymerase during the stationary growth phase (Wray and Fisher, 1993). In bacteria, it is common that genes have several promoters and different σ factors of RNA polymerase selectively recognize these promoters, to adjust the transcription pattern to environmental changes (Storz and Toledano, 1994). GlnR is considered to be a typical orphan response regulator since *glnR* has no co-localized gene encoding a putative sensory component (Fink et al., 2002). GlnR of *S. coelicolor* contains a conserved Asp residue (D-50) that could constitute an alternative potential phosphorylation site. In GlnR, the other two conserved sites are replaced by a Tyr residue corresponding to the OmpR T-83 and a Val residue (V-95) corresponding to the OmpR Y-102 (Stock et al., 1989). Recently, Lin et al. characterized the structure of the GlnR-dependent transcription activation complex (GlnR-TAC). They reported the cryo-EM structure of GlnR-TAC and a co-crystal structure of the C-terminal DNA-binding domain of GlnR (GlnR_DBD) (Shi et al., 2023). The GlnR-TAC includes RNA polymerase, GlnR, and a promoter containing four conserved GlnR binding sites. These structures elucidate that four GlnR_DBDs bind around the promoter DNA in a head-to-tail manner, and four N-terminal receiver domain of GlnRs (GlnR-RECs) are formed in a tetramer state. The tetramerization of GlnR-RECs coordinately bridge domains of RNAP core enzyme with GlnR_DBDs, promoting stabilization of GlnR-TACs (Shi et al., 2023).

A crystal structural analysis of the N-terminal region of GlnR of *A. mediterranei* U32 was performed by Wang et al. and this study revealed that the previously referred to as “phosphorylation pocket” was not conserved but replaced by an Arg-52 present in the β3-α3 loop (Lin et al., 2014). The potential phosphorylation residue Asp-50 is not phosphorylated but is involved in the homodimerization of GlnR. Additionally, Asp-50 is involved in charge interactions between the receiver domain and the highly conserved residues Arg-52 and Thr-9. These interactions are likely to play a crucial role to maintain the correct conformation for homodimerization (Lin et al., 2014) that affects GlnR DNA-binding capacity (Wang and Zhao, 2009). The receiver domain of the N-terminal end of GlnR forms a homodimer through the α4-β5-α5 dimer interface, while the aspartate residue at the N-terminal does not need to be phosphorylated (West and Stock, 2001). However, this site is necessary to maintain the stability of homodimerization (Lin et al., 2014).

In *M. smegmatis*, the aspartate residue D-48 is essential for GlnR activity under nitrogen limitation. When D-48 is changed to alanine, its phenotype is similar to that of a *glnR* mutant and showed blocked growth (Jenkins et al., 2012).

GlnRII, another regulator of nitrogen metabolism, is also an OmpR family protein whose C-terminal DNA domain is highly similar to that of GlnR (Fink et al., 2002). The α3-DNA recognition helix of GlnRII is almost identical to that of GlnR except for two amino acids Alanine (Ala-163 in GlnRII and Arg-195 in GlnR) and Arginine (Arg-167 in GlnRII and Ala-199 in GlnR) that may be related to the DNA binding specificity (Fink et al., 2002). This may explain why GlnR and GlnRII bind some common target genes. Interestingly, CheY, another regulator of the OmpR family, has α4 and β5 helixes (Fink et al., 2002) that are involved in the contact with the kinase CheA (Welch et al., 1998) whereas the N-terminal part of GlnRII does not contain such helixes (Fink et al., 2002). Moreover, GlnRII does not have the three conserved phosphorylation sites of OmpR family proteins, and it is not co-localized with a kinase (Fink et al., 2002). Therefore, the phosphorylation mode of GlnRII may differ from that of OmpR family proteins. As regulators of nitrogen metabolism, GlnR and GlnRII may receive different signals and have different target genes due to their structural differences. Since GlnR cannot be phosphorylated *in vitro* and its not co-localized with an histidine kinase, the post-translational modification of GlnR has been challenging to study. Amin et al. found that the transcriptional expression level of GlnR in *S. coelicolor* did not change significantly with nitrogen availability (Amin et al., 2016) whereas the transcriptional level of downstream target genes regulated by GlnR changes significantly according nitrogen source nature and availability. This indicated that GlnR binding activity might be modulated by post-translational modification. Indeed, it was demonstrated that GlnR is mainly phosphorylated on serine/threonine and acetylated on lysine (Amin et al., 2016). When nitrogen sources are abundant, GlnR is primarily modified by phosphorylation and cannot interact with the promoter region of its target genes whereas under nitrogen limitation, the acetylation of GlnR takes place but does not alter its DNA-binding capacity (Amin et al., 2016).

## 5 The conserved binding sites of GlnR

GlnR of *S. coelicolor* can bind to the *glnA* promoter regions of different Actinomycetes (Tiffert et al., 2008). Sequence comparisons of *glnA* promoter regions from various Actinomycetes including *S. coelicolor*, *S. avermitilis*, *Bifidobacterium longum*, *Frankia* sp. EAN1, *M. bovis*, *M. tuberculosis, C. glutamicum, Propionibacterium acnes, Rhodococcus* sp. RHA1*, Nocardia farcinica, Nocardioides* sp. JS614 showed that all Actinomycetes except *C. glutamicum*, whose nitrogen metabolism is regulated by AmtR, contain conserved GlnR binding sites (Tiffert et al., 2008). In *S. coelicolor*, the promoter sequences of the 13 GlnR target genes were compared, and the conserved GlnR binding sequence was determined to be gTnAc-n_6_-GaAAc-n_6_-GtnAC-n_6_-GAAAc-n_6_ (a-b-a-b), with each GlnR binding box having both a-site and b-site (Tiffert et al., 2008). Additionally, DNase I footprinting analysis accurately verifies the binding sequences on *glnA* and *gdhA* promoter regions. As predicted, two binding boxes were needed for GlnR binding in the *glnA* promoter region, while only one was necessary for GlnR binding in the *gdhA* promoter region (Tiffert et al., 2008). The GlnR binding box is frequently found on the coding strand of the target gene (Wang et al., 2012). However, there are exceptions. For example, SCO0888’s GlnR binding box is on the non-coding strand (Tiffert et al., 2008). Generally, when two GlnR binding boxes are present, they are continuous on the DNA sequence (Shi et al., 2023) but Wang et al. found that the two binding boxes of GlnR in the *nasA* promoter region were separated by 1 bp (Wang and Zhao, 2009). There are also three GlnR binding boxes on the promoter of the *amtB* gene, which is responsible for ammonium transport (Wang et al., 2012). A PhoP protected sequence also exists between the first and the second GlnR binding box (Wang et al., 2012). Wang et al. confirmed that the three binding boxes are necessary for *amtB* regulation by GlnR and revealed the molecular mechanism by which GlnR and PhoP cross-regulate *amtB* expression (Wang et al., 2012). Subsequently, Sola-landa et al. comprehensively analyzed the promoter regions of 14 GlnR target genes in *S. coelicolor*. They confirmed these targets by footprinting analysis, and proposed a novel 22-nt long consensus for GlnR DNA binding motifs (Sola-Landa et al., 2013). In this 22-nt long consensus, the second 11-nt long repeat is more highly conserved than the first one (Sola-Landa et al., 2013). In GlnR of *S. venezuelae*, a 16-bp GlnR box was proposed, which contained 5-bp a-site, 6-bp spacer and 5-bp highly conserved b-site (5 + 6 + 5-bp): GTnAC-n_6_-GTnAC (Pullan et al., 2011) but this consensus is fairly similar to the GlnR/22 nt long consensus. In addition, GlnR conserved binding sites were also identified in *M. smegmatis* (t/gTAAC-n_6_-Gc/aAAC) (Amon et al., 2008) and *S. erythraea* (t/gna/cAC-n_4_cn-GnAAc) (Yao et al., 2014) (Table 2). In *A. mediterranei* U32, the *nasACKBDEF* operon involved in nitrate assimilation (Shao et al., 2011), is directly regulated by GlnR (Wang Y. et al., 2013). Four GlnR binding sites were predicted by bioinformatics analysis in the promoter region of this operon (Wang Y. et al., 2013) but the studies revealed that only three sites (a1-b1-b2 sites) were necessary for GlnR binding: while a2-site was not required. EMSA (Electrophoretic Mobility Shift Assay) results demonstrated that GlnR yielded two binding bands with the *nas* promoter region forming two complexes (complexes I and II), where the complex with a lower mobility rate was designated complex II (Wang Y. et al., 2013). In this binding site pattern, a1-b1 sites were “5 + 6 + 5-bp,” just enough for the GlnR homodimer to combine and generate complex I. The generation of complex II required the b2-site. A 16-bp spacer between the b1-site and b2-site may cause a bend between the b1-site and b2-site upon GlnR binding as observed in other prokaryotic systems (PerezMartin and deLorenzo, 1997). Wang et al. determined the GlnR conserved DNA binding motif by changing the nucleotides of GlnR binding site on the *glnA* promoter in U32 one by one. This study revealed that the adenine at the fourth position was highly conserved and irreplaceable and proposed a 5-nt GlnR box as the fundamental motif for GlnR binding (Wang et al., 2015). Altogether these studies demonstrated that the GlnR binding mode is more adaptable than anticipated.

## 6 Summary and outlook

**Figure 3:**
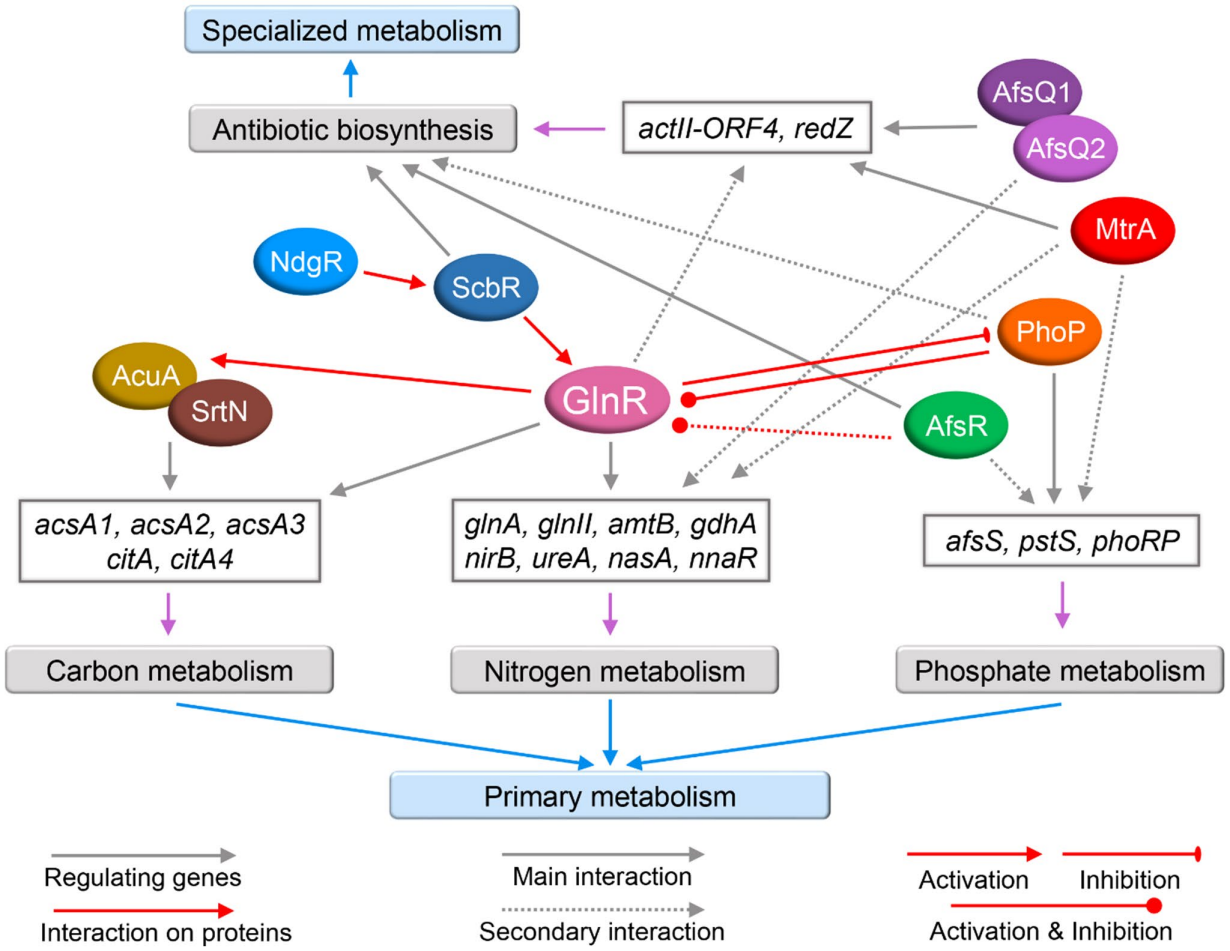
The cross-regulatory network of GlnR with other regulators in actinomycetes. The regulators, including GlnR, are represented by colored ellipses. Gray arrows represent certain proteins act as regulators for the expression of certain genes. Red arrows represent interactions at the protein level. Red triangle-headed arrows represent activation. Red bars represent inhibition. Red circular-headed arrows represent both activation and inhibition. Main interaction is indicated by solid lines and secondary interaction is indicated by dotted lines. The purple arrows indicate genes involved in the relevant nitrogen metabolism, phosphate metabolism, carbon metabolism, and antibiotic biosynthesis, and the corresponding genes are classified in gray boxes.

Under the stimulation of an unknown nitrogen signal(s), unknown sensory kinase(s) may transmit the signal(s) to GlnR, which then interacts with the promoter region of its target genes to regulate nitrogen metabolism. In addition, nitrogen metabolism is also cross-regulated by other regulators (Figure 3) such as PhoP, the regulatory protein of phosphate metabolism that can also control the expression of *glnR* and of its target genes belonging to nitrogen metabolism; MtrA and AfsQ1, which regulate antibiotic production but can also negatively regulate nitrogen metabolism, while AfsR, NdgR and ScbR positively regulate *glnR* expression. Genes and proteins in interaction with GlnR are summarized in Table 3. There are still many things to be revealed concerning GlnR regulatory role. Continuous research efforts should lead to the identification of the kinase(s) involved in the phosphorylation of GlnR as well as of the enzymes involved in its post-translational modification. The implementation of systems biology approaches including proteomics, transcriptomics, metabolomics, and bioinformatics will surely lead to the characterization of new direct and indirect GlnR target genes and novel cross-regulatory networks with other regulators.

**Table 3:** Genes and proteins interaction with GlnR in various metabolism.

	Protein	Gene
Nitrogen metabolism	NtrB/NtrC (ammonium assimilation); TnrA and GlnR^bsu^ (Regulate intracellular nitrogen availability) GlnR, GlnRII and AmtR (regulators in nitrogen metabolism) GlnE (adenylyltransferase) GlnK/GlnD NnaR (nitrate/nitrite assimilation) NasN (nitrate reductase) AmtB, Amt1 and AmtA (putative ammonium transporters) NarGHJI (nitrate reductase) NirBD (nitrite reductase) Alanine dehydrogenase (AlaDH)	*nrgAB*, *nasB*, *gabP*, *ureA*, and *glnRA* *glnR* and *glnRII* *glnA, glnA2*, *glnA3*, *glnA4*, and *glnII* *amtB, glnE*, *and nas* *glnQHMP*, *nasD1*, *nasD2EF, ansZ*, *pucR*, *gcvH*, *oppABC, dppABC*, and *appABCDF* *nirBD, nirB, nasA*, and *gdhA* *sco1863*, *sco2195*, *sco7155, sco0255*, *sco0888*, *sco2400*, and *sco2404* *nnaR* and *ald* *nasE, narK*, *nirA*, and *nasACKBDEF* *gltBD*, *gltP*, and *glnQ*
Carbon metabolism	non-PTS (non-phosphotransferase-system) carbohydrate ATP-binding cassette (ABC) transporter Agl3R ABC-type carbohydrate transporter AcsA1, AcsA2, and AcsA3 (acetyl-CoA synthetases) AcuA/SrtN (acetyltransferase/deacetylase)	*ldh3* and *maeA1* *agl3EFGXYZ* *icl and prpDBC* *citA* and *citA4* *gltA-2* *amlB, glaA*, and β-glucosidase-encoding gene *aglA2*, *aglA3*, *aglA4*, and *aglA5*
Specialized metabolites biosynthesis	LmrA (lincomycin transporter) RamR AflQ1-Q2 RifZ and RifK	*valK-valA-int* *actII-ORF4* and *redZ* *aveR* and *olmRI/RII* *rif* cluster *ectABCD*
Cross-regulation	PhoP/PhoR and AfsR MtrA AfsQ1/AfsQ2 AreB, NdgR and ScbR WhiB3, KstR	*afsS*, *pstS* and *phoRP* *actII-ORF4*, *redZ*, and *redD*

## Author contributions

BG: Investigation, Validation, Writing – original draft, Writing – review & editing. GL: Investigation, Validation, Writing – review & editing. DG: Funding acquisition, Validation, Writing – review & editing. JW: Conceptualization, Funding acquisition, Validation, Writing – review & editing.
